# "Immortal but frightened"-smoking adolescents' perceptions on smoking uptake and prevention

**DOI:** 10.1186/1471-2458-10-776

**Published:** 2010-12-21

**Authors:** Maria Nilsson, Maria Emmelin

**Affiliations:** 1 Dept of Public Health and Clinical Medicine, Epidemiology and Global Health, Umeå University, SE-901 87 Umeå, Sweden

## Abstract

**Background:**

To curb the tobacco epidemic a combination of comprehensive interventions are needed at different levels. Smoking uptake is a multi-factorial process that includes societal factors as well as social and individual characteristics. An understanding of the process is essential in order to model interventions. The aim of this study was to explore the role of smoking for young smokers by focusing on the mechanisms that facilitate young people starting to smoke as well as what could have prevented them from starting.

**Methods:**

A qualitative research design using focus group discussions was chosen as the basis for a content analysis approach. Eight focus groups were conducted with five to six participants in each (four groups with boys, four with girls). The informants were purposively selected to represent smokers in the age range of 15-16 years within the county. The total number of group participants was 44; 21 were girls and 23 boys. The study was performed at 7-9^th ^grade schools in Västerbotten County in northern Sweden.

**Results:**

Three themes related to different aspects of youth smoking behaviour emerged from the analysis. Theme 1) *"gaining control" *reflects what makes young people become smokers; theme 2) *"becoming a part of the self" *focuses on what facilitates youths to start smoking; theme 3) *"concerned adults make a difference" *indicates what may prevent them from starting.

**Conclusion:**

Young smokers described starting to smoke as a means of gaining control of feelings and situations during early adolescence. Smoking adolescents expect adults to intervene against smoking. Close relations with concerned adults could be a reason for less frequent smoking or trying to quit smoking. Interventions aimed at normative changes, with consistent messages from both schools and parents about the negative aspects of tobacco seem to be a feasible approach for preventing youth from using tobacco.

## Background

The tobacco pandemic calls for action on international, national and local levels. Preventing youth from smoking is a global challenge. Worldwide, about 80-100 000 young people become addicted to tobacco every day [1]. To curb the tobacco epidemic a combination of comprehensive interventions at different levels are needed. An understanding of the interaction within and between levels is a prerequisite for successful interventions.

For generations, tobacco industry marketing has filled smoking with values that make it attractive for young people regardless of cultural context. The marketing has targeted intra personal values that are important during the psychosocial development and socialisation processes of young people. Studies on youth tobacco uptake have shown that knowledge is not enough to prevent them from starting to smoke [2]. In a Swedish study, high levels of knowledge about risks did not predict future non-use of tobacco. The researchers concluded that attitudes and expectations may determine knowledge, rather than the other way around [3]. The young smoker becomes a smoker in a social context, not in a vacuum. Factors influencing the process from initiation to maintenance of regular smoking are both individual and contextual and intertwined in a complex interaction. The young person is an agent in his/her own life and has different smoking predictors on a person level. Families, peers and schools are agents influencing individual behaviours and social normative processes. The interrelationships between adolescent smoking and social and personal influences that are part of the adolescent developmental process are similar across countries [4]. To prevent youth tobacco use, bidirectional strategies are suggested. At the national level, legislation and regulations can create a broad societal influence that goes beyond individual and family influences, making processes normative. At the local level, intervention programs and strategies may have influence through integration of social, environmental and cultural factors.

The smoking prevalence in Swedish youth aged 15, has decreased since the 1970 s. Since the end of the 1990 s it was fairly stable but during the last years a slight increase in boys smoking was noted. The current smoking prevalence for boys was reported to be 23% and for girls it was 30% and the snus use prevalence 15% and 4% respectively. Thus, smoking was more prevalent then snus use (Swedish moist snuff), especially in girls, but taking snus use into account boys were more often a tobacco user [5].

In 1993-94, Sweden passed the first tobacco act that prohibited smoking on school premises. In 1997, the act was complemented with an age limit on tobacco sales and sales were not allowed to young people below the age of 18. In 2005, smoking was banned in restaurants, bars and cafés. Each of these laws was preceded by public debates and expression of opinions through national and local media.

One of the domains of the Swedish national public health policy focuses specifically on the use of addictive substances, including tobacco. An intermediate aim for 2014 is to halve the number of young people below the age of 18 that start to smoke or use snus [6]. To be able to reach this target, actions at the national level need to be supplemented by the introduction of locally and regionally developed intervention programs. Modelling these interventions is needed to build a deeper understanding of how and why health behaviours change during adolescence, why risk behaviour like smoking becomes attractive for the individual, and how tobacco uptake can be prevented. In this paper we present the results from a study in Västerbotten County on young people's views of smoking.

The overall aim of the study was to explore the role of smoking for young smokers by focusing on the mechanisms that facilitate young people to start smoking as well as what could have prevented them from starting.

## Methods

### Study area

The study was performed in the county of Västerbotten, situated in the north of Sweden. Västerbotten has 256 000 inhabitants living in 15 communities, an area close to 60 000 km^2^. There are fifty 7-9^th ^grade schools in the county and the settings are more rural in the inland and more urban by the coast. An intervention programme called Tobacco Free Duo has successfully been targeting adolescent tobacco use in the county since 1993 [7].

### Study design

Our main research interest was youth perceptions of smoking. A qualitative research design was used to reach an in-depth understanding of the youths' experiences, attitudes and beliefs as well as their wishes and concerns for the future. Focus group discussions were regarded the most appropriate method of data collection. The methodology builds on group interaction and is especially valuable for capturing how views are constructed and negotiated [8,9]. The group discussions were part of a larger study on smoking and gender with youth in five European countries.

### Sampling of informants

The informants were purposively selected to capture diversity with an aim of representing boy and girl smokers who were 15-16 years of age. We limited our study to smokers since non-smokers could only have reflected on what they believe about others. We considered schools to be the best source for recruitment since they provided a well-functioning network that allowed us to easily reach young smokers who were willing to participate. Thus, we selected four schools; three in urban settings representing areas with different socio-economic statuses, and one in a more rural area. Student social welfare staff, school nurses, teachers and youth club leaders helped distribute written information and an invitation letter to smoking youth. A smoker was defined as someone who smokes on a regular basis, at least once a week. This may have influenced the selection process and given us well-known adolescent smokers. However, the recruitment method varied and in some cases young people who had decided to participate brought additional participants from among their peers.

### Data collection

The first author (MN) performed the data collection over a period of two months. This was preceded by pilot sessions used for testing the research tools. All the discussions were conducted within the school setting during school hours. They were held privately in special rooms without any school staff present. To be able to evaluate gender differences, the groups were homogenous with regard to sex and there were five to six participants in each group. The discussion guide was thematic, flexible, and covered areas such as use of tobacco, perceptions, and tobacco-related attitudes and changes over time. Even though the school setting meant that the participants might know each other, this was not perceived as a limitation but as a positive factor to create a good discussion atmosphere. All discussions were tape recorded and the length varied between 55 to 90 minutes. The first interview was transcribed, preliminary analysis done, and discussed in the research group to allow for an emergent design with revisions and further elaborations in the forthcoming discussions. After additional three focus groups a new peer debriefing session took place to discuss and make decisions for further revisions in the data collection strategy.

In total eight focus group discussions were conducted, one girl- and one boy-group at each of the selected schools. The total number of participants in the groups was 44 out of which 21 were girls and 23 boys.

### Data analysis

**Figure 1:**
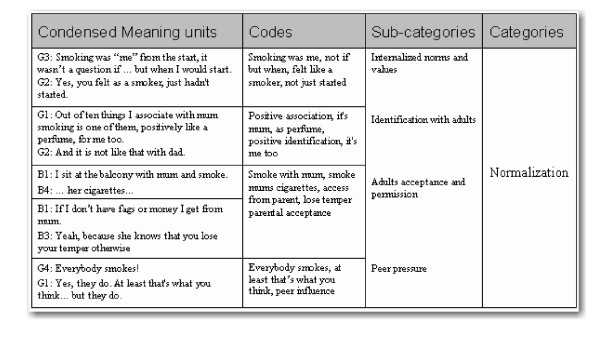
**An example from the analysis process**.

All focus group discussions were transcribed verbatim. A descriptive content analysis guided by Graneheim and Lundman was employed. Meaning units were identified, condensed and coded for creating categories, and themes describing both the manifest and the latent meaning were created [10]. Open Code software was used to facilitate the coding procedure [11]. An example of the analysis process is given in Figure 1, showing condensed meaning units with corresponding codes, sub-categories and a category.

Members of the research team were involved in the coding and analytical phase and contributed to the interpretation based on their differing professional expertise. Peer debriefing sessions were also used to help evaluate the main researcher's role in data collection and the analytical process.

### Ethical considerations

The school headmasters of each selected school sanctioned the study before students were approached. The school administration facilitated giving written and verbal information to parents and students about the aims of the study, its methodology, terms for volunteering, assurance of privacy, confidentiality in presentation of results, and the names and addresses of the responsible researchers. Since smoking is part of many teenagers' lives the research topic was not regarded as very sensitive. The participants had all reached an age (15-16) where they could make a mature and independent decision about their study participation. The study was approved by the Research Ethic Committee at Umeå University (dnr 02-251 § 270).

## Results

**Figure 2:**
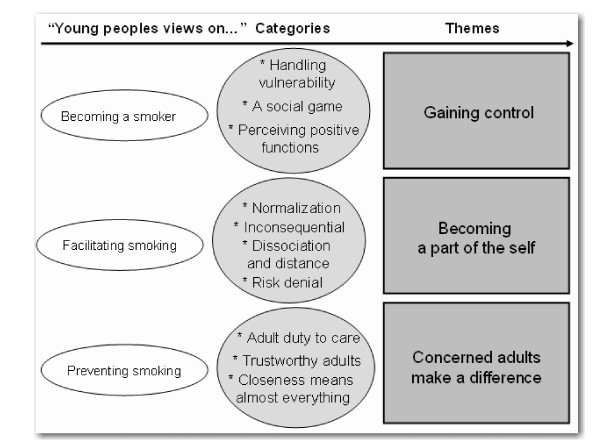
**Three themes of youth smoking**.

Based on the coding and the development of sub-categories and categories, three themes evolved that relate to different aspects of youth smoking behaviour as shown in Figure 2. The themes reflect young smokers' views on what makes young people become smokers, what facilitates youth to start smoking and what can prevent them from starting. The themes are presented in more detail below. The related categories are indicated in **bold **and illustrated by quotations to show how our interpretations are grounded in the focus group discussion data.

### Gaining control

Young smokers reflected on the process of becoming a smoker and described feelings that were both complex and contradictory; uncertainty about some aspects of life was combined with great certainty about others; feelings of curiosity and a wish to challenge existing norm systems were accompanied by feelings of fear, vulnerability and a need to comply with peer conceptions about attitude and image. Smoking was described as a short cut to **handling this vulnerability **and reaching social and adult status, making them feel more confident. During early smoking, cigarettes had the positive function of helping to build image and identity. One person called the cigarettes "my trademark". When reflecting on other smokers, early testers were described as having low self-esteem and thus a need for adopting something that would make them accepted and to belong to a group.

*G1: My God, you were so frightened...you were older, but more frightened*.

*G2: Yes, but when one starts the seventh grade, everything is new. You are the youngest at school...and you are supposed to be older and things like that. But you are so little, you know, and you need something to hold in your hand. Yes, well it can't be explained. Because the seventh grade was still bloody terrible. You're really a scared little scumbag that has to prove yourself to everybody all the time*.

Focus group no. 7

Our informants described smoking initiation as a **social game**. The first cigarettes were often part of an imitation of older friends, testing how to perform and inhale for later practice "in public".

*G1: When you first smoked you got dizzy and had to sit down*.

*G2: It was a guy who taught me to smoke...or take a deep drag. Anyway he had smoked for a long time... and he said that you never faint from a nicotine kick but you can throw up. So it was. Okay, I stood by the sink. So I stood there and leaned against the counter instead and a friend, she had to sit down on the floor and lean against the wall because it started spinning around so much*.

Focus group no. 5

Once they were established smokers, the young smokers described a culture of giving and getting cigarettes back. They spent a lot of time with other smokers at school and during spare time in what could be described as a "smoking community". The girls talked about smoking and sharing cigarettes as social putty while boys described it as nice having friends to smoke with. The description of themselves belonging to a "smoking community" with good friendships was common for girls and boys. The ones not being part of "the smoking community" according to young smokers meant the ones who were not "real" smokers; only smoking now and again and smoking on the sly. They described these younger ones, not belonging to their group, with a slight contemptuous attitude.

*B: Okay, they try to make it look like they are so terribly grown-up and experienced but they really are small, skinny young teens wearing short t-shirts with like bare stomachs. You don't respect them. They are the biggest fools in the world, who haven't understood anything, who try to be something they are not. Thinking that they are more than what they are*.

Focus group no. 8

The young smokers described several **perceived positive functions **of smoking with friendship being one of the most important. As part of the smoking community you spent lots of time together and made close friends. But the cigarette was also described as a loyal friend, something to pass time with and keep you occupied. The drug effect or "nico-kick" was perceived positively and described as a way to increase well-being. The girls described it as a way to handle stress and negative emotions.

### Becoming a part of the self

When reflecting on what facilitated the process of becoming a smoker, the young smokers described a **normalization **of smoking that for many started in early life. Almost all of the informants' parents were smokers and/or snus users.

*B: Nicotine really flows in our house, that's how much my parents smoke. Nicotine has flown in my veins since mama smoked when she was expecting me. It's my fate*.

Focus group no. 4

The study participants described many early memories that influenced them and most likely contributed to an internalization and identification as a smoker-to-be. When they were children, they described their parents smoking, observed that they relaxed and seemed to feel good, and perceived smoking as a natural part of their parents.

*G: I have grown up with mama smoking, it has always been that way. It was like normal--like I eat or mama smokes. So that's just the way it is. I don't think so much about it 'cause I'm so used to it. And that makes it okay for me, from her that I smoke, it's like the way it is. So if I would say ten things that I relate to mama then smoking would be one of them, but that's not at all bad. It's like you have a certain type of perfume. Smoking becomes part of you, something like Göran Persson (Sweden's former Prime Minister) and politics. It's about the same thing. And even if I don't think about it, it becomes like just a part of me too*.

Focus group no. 7

Parental smoking gave access to cigarettes. For many, the first cigarette smoked was a cigarette picked up secretly at home. Cigarettes from parents continued to be one of the main sources for many, either given or secretly taken. The provision of cigarettes at home was perceived as an open or hidden permission to smoke and contributed to the normalization of smoking. Many described acceleration in smoking when parents permitted, or even more when they smoked together with their children. The informants stated that when parents allowed their children to smoke the school lost its potential power to intervene against their smoking. It was stressed that smoking parents weakened their position in making their child smoke free. Getting hold of cigarettes outside their homes were described as easy; the word on where to buy cigarettes spread quickly or you could get or buy cigarettes from friends.

Another facilitating factor was the perceived peer influence/pressure emanating from the notion that everybody smoked, or at least all significant ones did. Older, nice friends were smoking role models or even introduced them to smoking.

*G: "Perhaps not always the coolest ones, but like the older friends who are okay and all that. And then it's that, that has been important. That they are okay and that they are smoking*.

Focus group no. 5

The informants expected adults to act against smoking but adults were often described as passive, doing nothing or more or less resigned. The majority of the young thought that significant adults like parents and teachers should intervene. *"It's what they should do; it's part of the package of being an adult"*. When they did not act it was regarded as acceptance and as facilitation. The young smokers knew that smoking was not allowed at the school, but they regularly saw school staff smoking on the school premises and not following the regulations themselves. Some gave examples of smoking school staff gossiping about what teachers were doing when no students were present. Both being examples of adults undermining trust and rules. Others described parents smoking on the sly as an example of undermining trust and respect. Informants said that when their mothers and fathers gave different messages and set different rules, they lost their chance to intervene. Another factor of **inconsequence **facilitating was also parents' cigarettes being available at home. Many youth thought it strange that parents said you should not smoke and then had no control of the cigarettes at home. *"They deny and supply at the same time"*. The young smokers felt that inconsequential and deceitful role models were dangerous.

Feelings of **dissociation and distance **within themselves as well as in relation to others accelerated smoking. They described distance as being part of life today*--"times have changed"--*with adults being busy, often lacking energy to intervene, and parents losing power in relation to their children. The informants stated that young people of today decide for themselves and that their own will and choice have to be accepted; they don't listen and they don't care. They also meant that parents were wrong intervening when they scolded them loudly and started conflicts about smoking, when they nagged or punished them. These actions resulted in obstinacy and the young smoking even more. Many informants experienced their parents giving up their children's smoking. The parents were irritated when teachers phoned home and perceived the telephone calls rather than the smoking to be the problem. They described their parents having *"zero check" *of what was going on, being disengaged, and without the possibility of exercising any influence.

Many youth disregarded smoking risks as a means of facilitating smoking. The knowledge of health risks was not homogenous; some seemed well informed while others were ignorant and made "logical somersaults" to facilitate continued smoking.

*G: I don't think that smoking causes illness. My mother hasn't smoked and my grandmother hasn't either, but she has cancer. Like skin cancer or breast cancer or something like that. And if you can get it without smoking why should I get it just because I smoke, since I could get it just as well without smoking? So like I see it, smoking doesn't have anything to do with it. Like neither mama nor grandma has smoked even once during their lives, like I really don't think they have tasted it at all. And anyway, if they got cancer, why should I get it? I don't think the chances are any bigger just because I smoke. I think I can get cancer just as well without smoking. So it doesn't make any difference*.

Focus group no. 1

**Risk denial **didn't seem to depend on level of knowledge. Expressions such as life being full of risks, being part of the package, "live hard and die young" were common. Many used myths of youth as a way to consciously underestimate and reduce risks. They found them comfortable to use as then they did not have to think about or stand up for their smoking.

### Concerned adults make a difference

When discussing prevention the young people constantly came back to the role of adults. On one hand, they often said that adults cannot do much about youth smoking. On the other hand, they listed what adults should and should not do, what they wanted from adults, and the possible impact. In general they emphasised their right to self-determination, including the right to smoke if they wanted to. Most of them said that their parents could not do much about their smoking. At the same time they stressed an **adult's duty to care **and used forcible words when they discussed adults that did not care. The young smokers expressed the view that parents had an assignment and an obligation to do all they could to support their children to not start smoking: "*It's a parental duty*". They found it tiresome if and when their parents "bothered" about their smoking. But a general expression was that the opposite would be worse and leave a feeling of being forgotten and not important. Common advice to parents was to act on suspicion and not be gullible, believing everything their children said. All the students had given false stories about their smoking to their parents when they first began smoking. A boy told his parents the following:

*B: Oh no! My friends smoke and I told them not to and they blew smoke at me*.

Focus group no. 3

All smoked during school hours and most of them did so at the school. They knew that smoking was not allowed within the school premises and shared the view that they expected teachers to intervene. As with their parents, they perceived it as a teacher's responsibility and expressed feelings like *"Good teachers care and then you respect them"*. Many expressed sympathetic feelings for the teachers having to intervene all the time. They thought it prevented smoking, especially during smoking initiation when one was not an established smoker and were still smoking on the sly. If the teachers' obligation to act was not there, it was perceived as that they did not care about the young smokers.

*B2: It might happen one out of a hundred times that a teacher tells you not to smoke at the schoolyard*.

B4: Comes up to you and says--"Now you have to..."

B1: "... go..."

*B2: And there are some teachers that go by and look the other way, since they are tired of saying something. Then there are others that say "How are things going?" And they just sit there beside you when you smoke. Some don't give a shit or act like they don't see. Somehow it feels strange that they don't even care. Then they can't really care about you in anything else either, it feels like that*.

Focus group no. 2

The young smokers reflected on the wish and need for **trustworthy adults**. That meant being consistent, expressing what they expected from the young, as well as giving relevant information and living up to their expectations of being a consequent role model. The young tended to lose respect when adults disappointed their expectations. An example given was *"Adult smoking on the sly sucks, it's pathetic"*.

The young smokers stated that the adults' attitude when they intervened determined the outcome. "Over-angry" or distant adults triggered defiance reactions. They asked for *"Respect please" *and when they felt respected, the young smokers were respectful in return. The young people talked about close relations, "***closeness meaning ****so much, **almost everything*****" **when it came to smoking. Close relations were important reasons to hide smoking, to smoke less and or try to quit smoking. Many told about loved grandparents not being aware of their grandchildren smoking and stated that the grandparents would be so disappointed if they knew. They expressed mixed feelings of not wanting to cause concern or worry, and feelings of being close to betraying trust.

When looking to the future, the informants reflected on being a parent themselves. At the same time as talking about themselves being a caring, observant, engaged parent not wanting their child to smoke, many realized the difficulties parental practices could bring.

*B: Then I get a call at home from the school and hear that my son smokes...Then I hang up the phone and go and lie down and pretend that I'm dreaming. Ugh! It must be so damn difficult having a child that smokes, when all you want is what is best for them*.

Focus group no. 2

## Discussion

In this qualitative study, young smokers reflected retrospectively on how they felt when starting to smoke, how those around them behaved and influenced them, and what could have made a difference. They described a complex and vulnerable time in life and emphasized several aspects of adults' roles and responsibilities in both facilitating smoking and preventing young people from starting to smoke.

Adolescence is a period of transition from childhood to early adulthood with an on going socialization process. The process includes learning and internalizing values; normative beliefs and behaviours are important to members of their social groups. Interpersonal skills and self-image are developed as part of early socialisation processes [12]. When the young smokers reflected on the process of becoming a smoker, they described feelings common for early adolescence such as uncertainty, vulnerability, and being afraid of not fitting in or being accepted by peers. Starting to smoke was a perceived means of controlling their feelings and the situation. This has implications for interventions aimed at preventing youth smoking. Interventions need to be directed toward both the individual and the school environment. There are programs with positive results on youth smoking that have used cognitive behavior and life skill modalities that address the individual [13]. In another study that focused on the school environment, researchers concluded that schools engaging and involving students in education and with good teacher-student relations also had lower smoking prevalence at school and were more effective [14]. Hendersen et al [15] specifically addressed gender differences in the effectiveness of school based interventions and reported that the quality of teacher-student relationships, student attitudes toward school, and the schools' focus on caring and inclusiveness could have an impact on smoking for both boys and girls aged 15-16 years. However, the effect was greater for male than for female students.

Both social status and adult status were stressed as important by study participants. Smoking has been given values attractive to adolescents through generations of tobacco industry marketing. These values contribute to the perceived social and adult statuses. These values continue to live on in the young generation even though tobacco ads have not been allowed in Sweden for several years. In a study of youth culture, Tilleczek [16] emphasised the need for considering these type of values when modelling interventions and to explore alternative ways to increase status with the adolescents.

Our informants described friendships in the "smoking community" as something that made them feel confident. The publicly shared identity as smokers was more important initially and followed later by feelings of closeness, solidarity and belonging. Thus, to prevent youth smoking, one has to understand the possible meanings and functions of smoking in young people's lives as well as that weak interpersonal skills and difficulty in social development may precede smoking uptake. Youth smoking cessation programmes have previously reported modest effects but there is mounting evidence of positive outcomes [17-19]. Development of youth smoking cessation programs could be improved by an increased understanding of the social dynamics of smoking. Young people could become lonely at school if they quit smoking. Programmes could improve by addressing how the feelings of friendship in the "smoking community" should be handled when quitting smoking. The drug effect from nicotine was positively described as a way to both increase well-being and handle negative emotions. This must be considered when designing youth cessation programmes.

The young people in this study described early testing of smoking. This pattern has been found in other studies of Swedish youth. Galanti et al found that as many as one out of five children reported having used tobacco by age 11 [20]. Olsson et al [21] studied changes in health behaviour in young people and reported that one in five girls and one in three boys aged seven to nine had tried cigarettes and snus. Girls seem to have a more rapid process of change when considering health behaviours including smoking and snus use. The onset of several behaviour changes was abrupt for both boys and girls and trying to smoke was one of them. Thus to prevent young people's use of tobacco, it is important to intervene before smoking becomes a part of their daily lives.

The youth in our study described the process of becoming a smoker as a normalization process starting early in life. They experienced parents and significant others smoking and had early memories that more or less made them identify themselves as smokers-to-be. Several participants said that "everybody" smokes. These results illustrate a process whereby both behavioural and normative beliefs forms attitudes, norms, intentions and behaviour as described in the theory of planned behaviour developed by Ajzen and Fishbein [22]. A young person is an agent in his/her own life with different predictors for smoking occurring on the individual level. But families, peers and schools are agents that influence the individual and social normative processes. Parents have a broad opportunity to influence their children's smoking. The lower children's perceptions are of effective parenting, the more likely they are to report tobacco use [23]. Parenting style and the quality of the relationship between the parent and child may affect the child's smoking [24-26]. In an American study, Tilson et al showed that high levels of parent-child connectedness could have a protective influence on youth smoking provided that the parent is a non-smoker [27].

Schools were described as "really dangerous environments" in relation to smoking during the discussions. In spite of the fact that smoking has not been allowed on Swedish school premises for many years, it is still a problem. In a previous national survey 82% of the 15 year old students said that pupils were smoking on their school grounds [28]. In this qualitative study participants talked about schools as one of the most influential areas for smoking initiation, escalation and development of regular smoking. The strong influence of school environment on youth smoking has also been observed by British researchers and the strongest influence was for the younger age groups. The researchers concluded that school culture is an independent risk factor for smoking [29-31].

Easy access to tobacco was an important factor that facilitated the process of becoming a smoker. This access can be interpreted as permission to smoke and provides positive, non-verbal smoking norms.

The adolescents expressed high expectations of parents, teachers and other significant adults. An expectation on parental action against children's smoking has been shown in a previous questionnaire study in Sweden [32]. When elaborating on factors that could prevent young people from smoking, they ask for caring, concerned, consistent and trustworthy adults. They wish to avoid big conflicts around smoking and stress the need of close and respectful relations. According to study participants, significant caring adults can make a difference.

## Conclusions

This study has several implications for prevention. Most importantly, smoking adolescents expect adults to intervene against their smoking and if they do not do so they are considered unconcerned. Thus, involving close, concerned adults in intervention programs might prevent and/or decrease adolescent tobacco use. Adults need to understand their significant role in young people's tobacco use. If they use tobacco themselves, they model tobacco use. Interventions aiming at normative changes with consistent messages from both parents and schools about the negative aspects of tobacco seem feasible approaches for preventing youth tobacco use. Concrete actions against smoking in the school yards are important to avoid schools as areas where smoking becomes established in young people's lives. Interventions should also focus on limiting general exposure and access to tobacco since it is clear that this signals an important normative message about the dangers of smoking.

## Competing interests

The authors declare that they have no competing interests.

## Authors' contributions

MN was the main author of the manuscript and involved in all aspects of the study. ME was a co author and provided scientific oversight and feedback throughout the development of the study and the manuscript. The co-author has seen and approved the final version of the paper and has agreed to its submission for publication.

## Pre-publication history

The pre-publication history for this paper can be accessed here:

http://www.biomedcentral.com/1471-2458/10/776/prepub
